# The ultimate preoperative C-reactive protein-to-albumin ratio is a prognostic factor for survival after pancreatic cancer resection

**DOI:** 10.1186/s40001-020-00444-z

**Published:** 2020-10-07

**Authors:** Laura van Wijk, Guus W. de Klein, Matthijs A. Kanters, Gijs A. Patijn, Joost M. Klaase

**Affiliations:** 1 Department of Hepatobiliary Surgery and Liver Transplantation, University Medical Center Groningen, Hanzeplein 1, PO Box 30001, Groningen, 9700 RB The Netherlands; 2 Department of Surgery, Isala, PO Box 10400, Dokter van Heesweg 2, Zwolle, 8000 GK The Netherlands

**Keywords:** Albumin, C-reactive protein, Albumin, CAR, Modified Glasgow Prognostic Score, Pancreatic cancer, Survival

## Abstract

**Background:**

Emerging evidence indicates that an elevated C-reactive protein-to-albumin ratio (CAR) may be associated with a poor prognosis in pancreatic ductal adenocarcinoma (PDAC). Further evidence showing that this ratio has significant prognostic value could contribute to current prediction models and clinical decision-making.

**Methods:**

Data were analysed of consecutive patients who underwent curative pancreatic resection between 2013 and 2018 and were histologically diagnosed with PDAC. We investigated the relation between the ultimate preoperative CAR and overall survival.

**Results:**

A total of 163 patients were analysed. Median overall survival was 18 months (IQR 9–36). Multivariate analysis demonstrated that a higher CAR (HR 1.745, *P* = 0.004), a higher age (HR 1.062, *P* < 0.001), male sex (HR 1.977, *P* = 0.001), poor differentiation grade (HR 2.812, *P* < 0.001), and positive para-aortic lymph node(s) (HR 4.489, *P* < 0.001) were associated with a lower overall survival. Furthermore, a CAR ≥ 0.2 was associated with decreased overall survival (16 vs. 26 months, *P* = 0.003).

**Conclusion:**

We demonstrated that an ultimate preoperative elevated CAR is an independent indicator of decreased overall survival after resection for PDAC. The preoperative CAR may be of additional value to the current prediction models.

## Introduction

Pancreatic cancer is the fourth leading cause of cancer-related deaths worldwide, with a 5-year survival rate of 9% for all stages combined [1]. For pancreatic tumours, surgical resection is the mainstay of treatment while (neo-)adjuvant therapy is gaining ground. Since morbidity and mortality rates after surgery are high, there is a need for identifying preoperative biomarkers that would enable better stratification of patients who may benefit from surgery.

In recent years, emerging evidence has shown the potential value of a variety of systemic inflammation-based prognostic scores in pancreatic cancer [2–7]. Serum elevation of C-reactive protein (CRP), an acute-phase protein, has been shown to be a prognostic indicator in a variety of neoplasms [8–11]. Moreover, hypoalbuminemia brought about by malnutrition and related to cachexia has been reported to be correlated with an unfavourable prognosis of gastrointestinal tumours [12, 13].

An elevated C-reactive protein-to-albumin ratio (CAR) or a composite score such as the modified Glasgow Prognostic Score (mGPS) seems to be potentially useful biomarkers for survival, but the evidence remains controversial [2, 3, 6, 14]. The mGPS combines the serum elevation of CRP and the decrease in albumin concentration, whereas the CAR is a continuous and more quantitative measure. Two recent meta-analyses showed that CAR was a useful prognostic factor of outcome in patients with pancreatic cancer; however, both studies included only studies in Asian populations [15, 16]. Furthermore, there is no consensus about the optimal cut-off value of CAR. The cut-off value of CAR ranged between 0.04 and 3.85 within the included studies in the meta-analysis from Zang et al. [16]. Currently, the most reliable prognostic factors for survival after PDAC are tumour size, lymph node status, resection margin and differentiation grade [17]. However, these prognostic factors rely on surgical exploration [5]. Further evidence demonstrating that the CAR can predict survival may contribute to current prediction models and support clinical (shared) decision-making. The aim of our study was to investigate the prognostic value of the ultimate preoperative CAR and the optimal cut-off value after resection for PDAC as compared with several established prognostic factors.

## Methods

### Patients

Between January 2013 and December 2018, all consecutive patients who underwent pancreatic resection and were pathologically diagnosed with PDAC at the University Medical Centre Groningen, the Netherlands, or the Isala clinics, the Netherlands, were included in the present study. All medical records were retrospectively reviewed. Patients were excluded if data relating to their preoperative CRP or albumin were missing or if they already had metastatic disease at the time of resection. All patients were followed up until October 2019 or death. Survival status was assured using the national Personal Records Database. This study was approved by the Institutional Review Boards of the University Medical Centre Groningen and Isala Zwolle (research registration number: 201900699).

### Data collection

Baseline characteristics were collected from the electronic medical record system. Laboratory tests were routinely conducted for each patient preoperatively. The laboratory results closest to the date of surgery were used for analysis. The following laboratory tests were conducted: CA 19-9, CEA, haemoglobin, bilirubin, CRP and albumin. The CAR was calculated by dividing the serum-CRP level by the serum-albumin level [2, 3]. The mGPS was calculated according to the following method: patients with an albumin level greater than 35 g/L and a CRP level less than < 10 mg/L were scored 0; patients with only an elevated CRP (> 1 mg/dL) were scored 1; and patients with low albumin (< 3.5 g/dL) and high CRP (> 1 mg/dL) were scored 2 [18]. Patients’ preoperative physical performance was determined according to the Eastern Cooperative Oncology Group (ECOG) [19] scale and the ASA-score. The type of pancreatic resection was selected based on tumour location and was classified into two groups: pancreatic head resections (pylorus-preserving pancreatoduodenectomy, or Whipple procedure) and other types of pancreatic resection (distal pancreatectomy, central pancreatectomy, total pancreatectomy). Postoperative complications were categorised into minor (Clavien–Dindo 1–2) and major complications (Clavien–Dindo 3–5). Overall survival time was defined as the time between date of surgery and date of the final follow-up or date of death.

### Statistical analysis

Discrete variables were described as total and percentage, and continuous variables as median and interquartile range (IQR). The primary outcome was overall survival after pancreatic resection with curative intention. Univariate Cox regression was used to identify possible prognostic factors (i.e., when the *P* value was below 0.1). These variables, along with known prognostic factors in pancreatic cancer, were included in a stepwise multivariate Cox proportional-hazard regression analysis to ascertain independent prognostic factors. For the CAR, the optimal cut-off point was estimated with a receiver operating characteristic (ROC) curve using Youden’s index. The resulting bivariate variable (high or low ratio) was also tested for prognostic value in overall survival. Additionally, baseline and clinicopathological characteristics were tested on difference in patients with low and high CAR (Chi-square test, Fischer’s exact test, Mann–Whitney *U* test, as appropriate). *P* values under the significance level of 0.05 were considered significant. For all statistical analyses, SPSS version 24 (IBM, Armonk, NY) was used.

## Results

### Study population

**Fig. 1:**
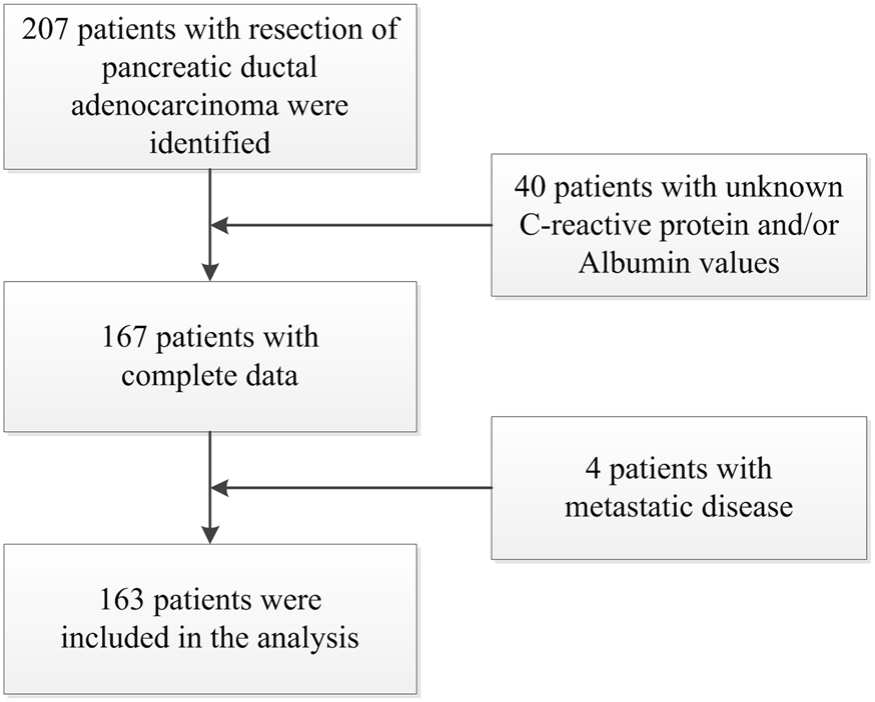
Flowchart of patient inclusion *PDAC* pancreatic ductal adenocarcinoma, *CRP* C-reactive protein

A total of 207 patients underwent resection of histologically confirmed PDAC at our institutes from 2013 to 2018. In 40 patients, CRP or albumin were not determined preoperatively. Additionally, four patients were retrospectively found to have metastatic disease at the time of resection (two pulmonary, one hepatic, and one omental metastasis). This left 163 individuals resected with curative intent for our study population (Fig. 1).

### Baseline and clinicopathological characteristics

Baseline and clinicopathological characteristics are presented in Table 1. Postoperative major morbidity occurred in 26 patients (16%), and the mortality rates within 30 and 90 days were one (0.6%) and nine (5.5%), respectively. Differences in baseline and clinicopathological characteristics between patients with low (< 0.2) and high CAR (≥ 0.2) are also presented in Table 1. Mean haemoglobin was lower in patients with high CAR (*P *< 0.001), and patients with high CAR had a higher metastatic lymph node ratio. Although the type of resection appeared to be different between patients with high and low CAR, when grouping the resections into pancreatoduodenectomy (pancreas-head tumours) and other pancreatic resections, no significant difference was observed (*P* = 0.112).

**Table 1:** Baseline and clinicopathological characteristics in relation to CAR with cut-off at 0.2. Percentages represent proportion within group

	Total *n* = 163	CAR < 0.2 *n* = 90 (55%)	CAR ≥ 0.2 *n* = 73 (45%)	*P* value
Sex				0.875
Male	87	49 (56%)	38 (44%)	
Female	76	41(54%)	35 (46%)	
Age, years (mean, SD)	66 (± 9.7)	65 (± 9.7)	67 (± 9.7)	0.591
ASA				0.055
I	12	7 (58%)	5 (42%)	
II	121	73 (60%)	48 (40%)	
III	29	10 (34%)	19 (66%)	
IV	1	0 (0%)	1 (100%)	
ECOG grade	*11 unknown*	45 (59%)	31 (41%)	0.380
0	76	29 (52%)	27 (48%)	
1	56	7 (50%)	7 (50%)	
2	14	1 (20%)	4 (80%)	
3	5	1 (100%)	0 (0%)	
4	1			
Haemoglobin, g/dL (mean, SD)	12.9 (± 1.6)	13.4 (± 1.5)	12.1 (± 1.6)	< 0.001
CEA, ng/ml (median, IQR)	4.1 (2.2–6.7)	4.5 (2.2–6.6)	3.2 (2.2–7.5)	0.577
CA 19-9, U/ml (median, IQR)	246 (60–936)	342 (54–867)	133 (62–1092)	0.800
Supplementary nutrition	*38 unknown*			
No	61	39 (64%)	22 (36%)	0.398
Enteral	57	31 (54%)	26 (46%)	
Parenteral	7	3 (43%)	4 (57%)	
Neoadjuvant therapy				0.692
No	157	86 (55%)	71 (45%)	
Yes	6	4 (67%)	2 (33%)	
Approach				
Open or conversion	154	85 (55%)	69 (45%)	1.000
Laparoscopy	9	5 (56%)	4 (44%)	
Type of resection				
PPPD	106	59 (56%)	47 (44%)	0.043^a^
PD (Whipple’s)	24	9 (37%)	15 (63%)	
Distal pancreas resection	22	17 (77%)	5 (23%)	
Central pancreas resection	2	0 (0%)	2 (100%)	
Total pancreatectomy	9	5 (56%)	4 (44%)	
Complication				
Clavien–Dindo 0–2	137	76 (55%)	61 (45%)	1.000
Clavien–Dindo 3–5	26	14 (54%)	12 (46%)	
Tumour size in mm (median, IQR)	30 (25–40)	30 (25–40)	35 (25–40)	0.477
Differentiation grade	25 unknown			
Well or moderate	88	49 (56%)	39 (44%)	0.861
Poorly	50	27 (54%)	23 (46%)	
Metastatic lymph nodes				
< 5	108	64 (59%)	44 (41%)	0.183
≥ 5	55	26 (47%)	29 (53%)	
Metastatic lymph node ratio	0.16 (0.06–0.26)	0.13 (0.04–0.25)	0.19 (0.01–0.29)	0.007
Metastatic lymph node ratio				
0	36	24 (67%)	12 (33%)	0.005
< 0.10	26	20 (77%)	6 (23%)	
≥ 0.10	101	46 (45%)	55 (55%)	
Para-aortic lymph node				
No metastasis	152	85 (56%)	67 (44%)	0.543
One or more metastases	11	5 (45%)	6 (55%)	
Radicality	*2 unknown*			
R0	94	53 (56%)	41 (44%)	0.726
R1	64	35 (55%)	39 (45%)	
R2	3	1 (33%)	2 (67%)	
Adjuvant therapy	*5 unknown*			
No	56	28 (50%)	28 (50%)	0.246
Yes	102	61 (60%)	41 (40%)	

*ASA* American Society of Anesthesiologists, *ECOG* Eastern Cooperative Oncology Group scale of performance, *CEA* carcinoembryonic antigen, *CA 19-9* carbohydrate antigen 19-9, *PPPD* pylorus-preserving pancreatoduodenectomy, *PD* pancreatoduodenectomy, *SD* standard deviation, *IQR* interquartile range

^a^pancreatoduodenectomy vs. other pancreas resections: *p* = 0.171

### Univariate and multivariate analyses using Cox multiple regression for overall survival

Median overall survival was 18 months (IQR 9–36) in the study population. Univariate Cox proportional-hazard regression was used to identify variables that were possibly associated with overall survival (Table 2). Stepwise multivariate Cox regression was performed using the variables sex, age, ECOG performance grade, haemoglobin, CAR, neo-adjuvant therapy, type of resection, tumour size, tumour differentiation grade, metastatic lymph node ratio, para-aortic lymph node status, and radicality. The ultimate proportional-hazard model was significant (*P* < 0.001) and consisted of sex, age, CAR, differentiation grade, and para-aortic lymph node status. A higher CAR was independently associated with lower survival; the hazard ratio was 1.745 (95% CI 1.200–2.539, *P *= 0.004). Due to significant collinearity with CRP and albumin, haemoglobin level was analysed separately, and stepwise Cox regression demonstrated that haemoglobin was not significantly associated with survival. Furthermore, when analysing CRP and albumin separately in multivariate analysis, only CRP was independently associated with survival (HR 1.006, 95% CI 1.006–1.027, *P* = 0.002). Additionally, when replacing the CAR by the mGPS, the variable mGPS ended in the ultimate regression model, but was non-significant (*P* = 0.077).

**Table 2:** Univariate and multivariate analyses using Cox multiple regression for overall survival. Variables presented under *multivariate analysis* represent the final model after stepwise exclusion

Covariate	Univariate analysis			Multivariate analysis		
	HR	95% CI	*P* value	HR	95% CI	*P* value
Sex						
Female	1			1		
Male	0.632	0.432–0.923	0.018^a^	1.977	1.191–3.282	0.001
Age (years)	1.034	1.011–1.059	0.004^a^	1.062	1.030 –1.094	< 0.001
ASA						
I–II	1					
III–IV	1.374	0.923–2.044	0.117			
ECOG grade						
0–1	1					
2–4	1.634	0.971–2.748	0.064^a^			
Haemoglobin	0.961	0.805–1.148	0.661			
Bilirubin	1.000	0.999–1.001	0.833^b^			
CRP	1.011	1.002–1.020	0.022			
Albumin	0.952	0.920–0.986	0.006			
CAR	1.406	1.038–1.905	0.028^a^	1.745	1.200–2.539	0.004
mGPS						
0	1					
1	1.419	0.946–2.128	0.090			
2	1.953	0.971–3.929	0.061			
CEA	1.011	0.999–1.022	0.074			
CA 19.9	1.215	0.940–1.570	0.138			
Supplementary nutrition						
No	1					
Enteral	1.264	0.838–1.905	0.264			
Parenteral	1.612	0.588–3.789	0.272			
Neoadjuvant therapy						
No	1					
Yes	0.541	0.171–1.710	0.296^b^			
Approach						
Open or conversion	1					
Laparoscopy	0.937	0.380–2.306	0.887			
Type resection						
Pancreas head	1					
Pancreas other	1.187	0.756–1.865	0.456 ^b^			
Complication						
Clavien–Dindo 0-2	1					
Clavien–Dindo 3-5	1.266	0.778–2.059	0.342			
Tumour size	1.007	0.996–1.018	0.237 ^b^			
Differentiation grade						
Well or moderate	1			1		
Poorly	1.271	0.837–1.931	0.261^b^	2.812	1.627–4.861	< 0.001
Metastatic lymph node ratio	2.946	1.154–7.521	0.024^a^			
Para-aortic lymph node						
No metastasis				1		
One or more metastases	3.299	1.738–6.262	< 0.001^a^	4.489	1.883–10.702	< 0.001
Radicality						
R0	1					
R1–R2	1.386	0.952–2.018	0.089^a^			

*ASA* American Society of Anesthesiologists, *ECOG* Eastern Cooperative Oncology Group scale of performance, *CEA* carcinoembryonic antigen, *CA 19-9* carbohydrate antigen 19-9

^a^Included in stepwise Cox regression because *p* < 0.1; ^b^Included in stepwise Cox regression because of prognostic value in the literature

### Determination of the cut-off point for the CAR

The mean CAR was 0.38 (SD 0.54); the median CAR was 0.16 (IQR 0.07–0.42). The optimal cut-off for the CAR ratio in predicting overall mortality was estimated using a ROC curve. Maximum sensitivity and specificity were found at a ratio of 0.2, which corresponded with a sensitivity of 54% and a specificity 69%, a positive predictive value of 78%, and a negative predictive value of 42% (Table 3).

**Table 3:** Number of patients who did survive and did not survive in relation to high and low C-reactive protein-to-albumin ratio (CAR) with a cut-off value of 0.2

	CAR < 0.2	CAR ≥ 0.2	Total
Alive	35	15	50
Deceased	55	58	113
Total	90	73	163

### Overall survival

**Fig. 2:**
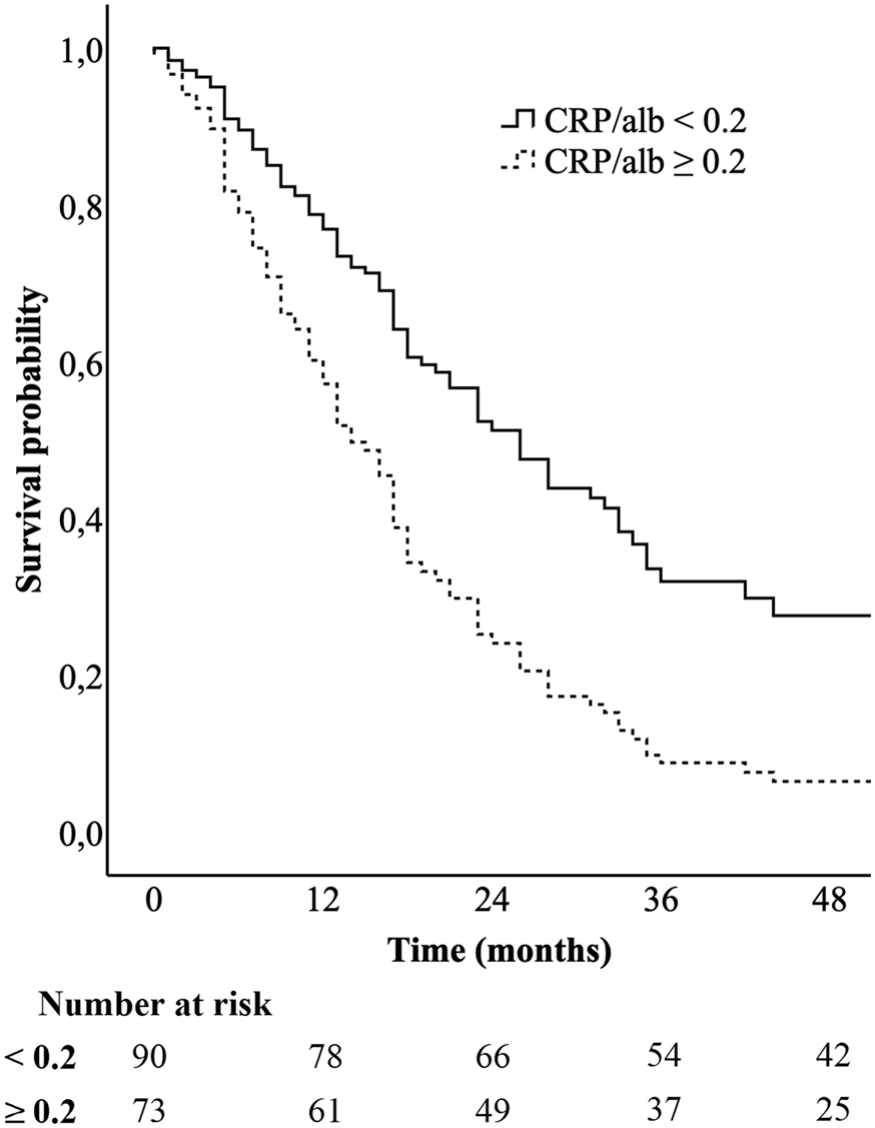
Overall survival of patients with low (< 0.2) and high (≥ 0.2) C-reactive protein-to-albumin ratios, corrected for age, sex, differentiation grade, and positive para-aortic lymph nodes using Cox regression

Median overall survival in patients with a low CAR was 26 months (IQR 11–56), and in patients with a high CAR 16 months (IQR 7–24). The final model using a bivariate variable of low (< 0.2) and high (≥ 0.2) CAR revealed that this variable was an independent prognostic factor as well (*P* < 0.001, HR 2.129, 95% CI 1.395–3.251). Survival was lower in patients with a high CAR (Fig. 2).

## Discussion

Our results demonstrated that a higher CAR together with a higher age, male sex, poor differentiation grade and positive para-aortic lymph node(s) was associated with a lower overall survival. A CAR above 0.2 is associated with a decreased overall survival in patients with PDAC after curative pancreatic resection. Corresponding with the previous literature, in our study, the CAR was an independent prognostic factor for overall survival in patients resected for PDAC [15, 16]. In addition, previous studies have shown the prognostic value of the mGPS on overall survival of patients with PDAC [20–22]. In our cohort, however, the mGPS was not an independent prognostic factor for overall survival, which was consistent with some previous studies [6, 14] although a prognostic trend was present. This might indicate that the CAR, being a continuous variable, may be a superior predictor if it is not condensed into a score or cut-off level. Therefore, we used the CAR as a continuous variable in the multivariate analysis. Doing so, we were more able to show a correlation between CAR and survival: the higher the ratio, the worse the predicted survival. In our cohort, maximum sensitivity (54%) and specificity (69%) were found at a CAR of 0.2. However, this result should be interpreted with caution since cut-off levels are rather heterogeneous in the literature. In a recent meta-analysis aiming to determine the potential role of CAR as a prognostic indicator in pancreatic cancer, the cut-off value of CAR ranged between 0.04 and 3.85 within the included studies [16]. Furthermore, both high CRP and low albumin were associated with poor survival, but only CRP was an independent prognostic factor for overall survival, indicating that the prognostic value of CAR is mainly driven by CRP. There is increasing understanding of the mechanism of the relation between the CAR and survival in patients with cancer. C-reactive protein is a marker of inflammation, and an elevated serum level might be caused by tumour necrosis or local tissue damage [9]. In addition, an elevated CRP could be a marker for a beneficial environment for the origin and growth of metastases. An elevated CRP gives an upregulation of the vascular endothelial growth factor, which promotes the growth and proliferation of tumours [7]. In addition, CRP is produced in response to elevated interleukin-6 levels [8]. Interleukin-6 promotes tumour growth by inducing multiple signalling pathways, including proliferation, angiogenesis and metabolism [23]. Hypoalbuminemia is often thought to reflect malnutrition in patients. However, emerging evidence shows that a low albumin level may also be a reflection of an inflammatory state [24]. The exact cause of low albumin levels in patients with cancer is unclear. The literature suggests that it is a combination of several mechanisms. One explanation is that high interleukin-6 levels produced by cancer cells inhibit the synthesis of albumin [25]. Alternatively, it may be the result of an increase in vascular permeability, which causes a redistribution of albumin, leading to lower serum levels and high extra vascular fluid levels [26, 27]. In accordance with the literature, men had a lower overall survival than women did [28–30]. It is well known that pancreatic cancer occurs more frequently in men. The underlying cause remains unclear. Possible explanations include differences in environmental or occupational risk factors, but other lifestyle factors, such as heavy smoking and high alcohol intake in men, may also contribute [31]. Alternatively, undiscovered genetic factors may play a role. These possible factors were assumed to also contribute to a higher mortality risk. In a recent review of clinical prediction models for survival after pancreatic cancer surgery, it was found that tumour size, lymph node status, resection margin and differentiation grade were most often included in the final prediction models [17]. In this study, all these variables were analysed, and the multivariate analysis showed that, of these variables, only differentiation grade and para-aortic lymph node status were significantly associated with overall survival. In the same review, it was also suggested to include neo-adjuvant therapy in the analyses. In our study, neo-adjuvant therapy had no significant predictive value, probably due to the small number (*n* = 6) of patients receiving neo-adjuvant therapy. However, the role of neo-adjuvant therapy is currently being investigated in the PREOPANC II trial and the CAR in these patients could be the subject of research in the near future. Moreover, Strijker and others have recommended to include the location of the tumour in the pancreas as a variable since previous studies have demonstrated differences in tumour biology between tumours in the head and corpus/tail [32, 33]. In our study, no statistical difference in overall survival was observed between head and distal pancreatic resections. The authors of the review have also commented that to objectively predict the outcome for pancreatic tumours, a distinction between different types of pancreatic and periampullary tumours should be made. Our study had several important strengths: we included only PDACs; we made a distinction between tumour locations; and we confirmed patients’ survival status using the national Personal Records Database. Our study was limited, however, by its retrospective nature, which among other consequences, resulted in the limited availability of laboratory results and confounding factors like preoperative pancreatitis, cholangitis or biliary drainage. Since biliary drainage might influence CRP, it may have been appropriate to include this variable. We did, however, include in the analyses the bilirubin level, which had no significant association with overall survival and did not influence the outcome. Over the last decades, variables used to assess the immune system and inflammation have gained interest as prognostic biomarkers for the prediction of outcomes for pancreatic cancer [2–7]. Since immunotherapy may play an important role in the future treatment of pancreatic cancer, our study and future research concerning pre-treatment prognostic (systemic inflammatory) variables could be of significant value [34]. Other possible reliable pre-treatment prognostic factors of outcomes for pancreatic cancer, besides CAR, are sex [28–30], CA 19-9 [17], LDH [35], age [17], tumour location [32], tumour size [36] and imaging texture features of the pancreatic tumour [37, 38]. Future research should focus on the development of a prediction model which includes pre-treatment prognostic factors. A prediction model based on pre-treatment parameters is essential for optimal patient management, based on informed shared decision-making processes, clinical trial design and interpretation of results. In conclusion, this study showed that an elevated ultimate CAR was independently and significantly associated with decreased overall survival in patients with PDAC after pancreatic resection. The CAR may, therefore, be of additional value to current prediction models and may be helpful in clinical decision-making.

## Data Availability

The datasets used and/or analysed during the current study are available from the corresponding author on reasonable request.
